# ConvGeM-next: a deep learning framework for plant disease detection

**DOI:** 10.3389/fpls.2026.1763739

**Published:** 2026-05-12

**Authors:** Zoya Arshad, Ali Javed, Abdul Khader Jilani Saudagar

**Affiliations:** 1 Department of Software Engineering, University of Engineering and Technology-Taxila, Taxila, Pakistan; 2 Information Systems Department, College of Computer and Information Sciences, Imam Mohammad Ibn Saud Islamic University (IMSIU), Riyadh, Saudi Arabia

**Keywords:** agricultural automation, ConvNeXt, deep learning, generalized mean pooling, plant disease detection

## Abstract

**Introduction:**

Plant diseases pose a major challenge to sustainable agriculture, particularly in regions that heavily depend on farming. Early and accurate identification of plant diseases is crucial for ensuring food production and minimizing crop losses. The rapid advancement of deep learning, particularly in convolutional neural networks (CNNs), has significantly enhanced plant disease classification performance. However, many models often struggle to generalize effectively in real-world scenarios due to challenges such as low-intensity visuals, low contrast between the background and foreground of the suspected sample, noise, and chrominance variation.

**Methods:**

To address the challenges mentioned above, we introduce ConvGem-NeXt, an end-to-end deep learning architecture specifically designed for fine-grained plant disease classification, built on the ConvNeXt baseline model featuring enhanced generalization capabilities. More precisely, our method incorporates a learnable Generalized Mean pooling layer and ReLU activation in the ConvNeXt model to enhance spatial feature representation, and a custom classifier head that integrates batch normalization, ReLU activation, and dropout to mitigate overfitting and improve classification accuracy.

**Results:**

We tested the presented model on two large-scale and diverse databases, PlantVillage and the PlantDoc. The model achieved 99.65% accuracy on the PlantVillage dataset and 94.69% accuracy on the real-world PlantDoc dataset, demonstrating the efficacy of our method for reliably classifying plant diseases.

**Discussion:**

This work contributes to the rapidly growing field of agricultural automation by providing a reliable framework for timely disease diagnosis and supporting the enhancement of crop productivity.

## 1 Introduction

Agriculture, which supports most developing economies, is crucial for sustaining livelihoods, national GDP, and environmental sustainability. Nevertheless, the emergence and spread of plant pathology are also one of the significant threats to the agricultural systems, leading to reduced crop yields, considerable losses, and food supply chain disruptions. According to the Food and Agriculture Organization (FAO) of the United Nations, it is estimated that by 2050, the world’s population will grow to over 9.7 billion, and this will increase the need for food production in the world to ensure food security (Lewandowski et al., 2017). The necessity to meet this demand is also becoming constrained by the decreased arable land, water scarcity, and crop diseases. In some developing countries, the annual yield alone has been observed to suffer a loss of up to 40% due to plant diseases alone, which poses a major setback to food security (Ahmed and Yadav, 2023; Shoaib et al., 2023). Thus, timely identification of plant diseases is important to reduce losses in yield. Traditional manual inspection is still common, especially in regions where modern technologies have not been fully available yet, but it is often inconsistent, time-consuming, and prone to error (Yilmaz et al., 2025). Laboratory tests that provide accurate results are microscopy and molecular diagnostics, which are extremely costly and not accessible to smallholder farmers (Sahu et al., 2022). The diseases of plants interfere with the physiological functioning, reducing the yield and quality, and they tend to show different symptoms, making it hard to detect them early. Further, visible symptoms such as lesions or spots on leaves or other discolorations may be indicative of infection, whereas in other plants, infection may occur without any visible effects until a large amount of destruction has occurred (Pacal et al., 2024). This subjectivity and dependence on trained personnel slow down interventions and bring financial costs to resource-constrained farmers (Sunil et al., 2023). All these issues demand scalable and dependable automated plant disease detection systems without undergoing any considerable reliance on expert help.

The agriculture industry has been employing Artificial intelligence (AI) based solutions to help produce more crops through early identification of plant diseases and pests. The AI application based on deep learning (DL) and computer vision (CV) has ensured that plant diseases can be monitored in a proper and non-invasive manner (Pacal et al., 2024), since it provides automated systems that can mitigate subjectivity and enhance the speed and accuracy of the diagnostic process. DL approaches outperform traditional machine learning (ML) methods because they automatically capture fine hierarchical patterns in raw images, enabling more precise detection, unlike conventional ML approaches that rely on manually crafted features like color, texture, shape, etc. (Nawaz et al., 2022). Combined, these methods not only offer high diagnostic accuracy but also scalability, which may be applied to track disease at the small scale and crops at the large scale. Mobile devices and drones have been used to expand access to AI-driven solutions, as well as sensing that are both cheap and made available even in resource-constrained settings (Albattah et al., 2023). These online technologies and intelligent algorithms are gradually transforming the nature of agricultural monitoring in the form of early warning, interventions, and environmentally friendly actions.

Regardless of this progress, the issue of transferring CNN models between controlled research datasets and field settings is still a significant issue. Most of the available models are trained using datasets, like PlantVillage (Mohanty et al., 2016), comprised of clean and standardized images that are easily learnable. Although these datasets are feasible in benchmarking, they lack representativeness of variability in the real world, such as in illumination, cluttered backgrounds, overlapping leaves, and even morphological differences among plant species (Abade et al., 2021). The models designed in the laboratory, thus, are unlikely to be very helpful in real life, making it impossible to scale to mass deployment. To overcome this gap, it is necessary to design architectures that retain the effectiveness of CNNs and increase the flexibility to deal with the heterogeneous field conditions and various crops. This has led researchers to explore new models that combine convolutional strengths with innovations inspired by transformers, giving rise to models such as ConvNeXt that aim to deliver both accuracy and robustness under real-world constraints. ConvNeXt model, based on design ideas of Vision Transformers, has demonstrated good performance in image recognition tasks due to its scalable design, uniform generalization, and integration of layer normalization, GELU activation, and depth-wise convolutions (Lewandowski et al., 2017). Nevertheless, to make it more suitable for plant disease detection in the real field, it requires some adjustments to enhance the resistance to environmental factors. This research suggests an end-to-end DL architecture, ConvGem-Next, by introducing specific architectural refinements to the base ConvNeXt architecture, enabling our method to capture disease-specific patterns in different conditions and plant growth stages. The main contributions of this work are as follows:

1. We present ConvGem-Next, an enhanced ConvNeXt model built with redesigned convolutional blocks and a custom classification head for reliable detection of different plant diseases.
2. We introduce the Generalized Mean pooling (GMP) layer within convolutional blocks to enhance feature representation and significantly improve classification accuracy for plant disease detection.
3. We introduce a customized classification head that includes a linear layer, batch normalization, Rectified Linear Unit (ReLU) activation, dropout, and a final linear layer to improve regularization, mitigate overfitting, and strengthen the model’s generalization capability.
4. We employ ReLU activation to maintain sparsity and computational efficiency while ensuring stable gradient propagation.
5. We conduct comprehensive evaluations on both benchmark datasets and real-world imagery, demonstrating improved generalizability and reduced overfitting compared to baseline models.

The remaining study is organized as follows: Section 2 reviews the related work on plant disease detection. Section 3 presents the details of our proposed ConvNeXt-based architecture. Section 4 outlines the details related to results and evaluation, and the conclusion is presented in Section 5.

## 2 Related work

In this section, we investigated the existing approaches to plant disease detection, with a special focus on the evolution of techniques from traditional machine learning to modern deep learning approaches. The prior work is categorized into two major segments: conventional ML-based techniques and DL-based frameworks.

The use of handcrafted features and classifiers like Support Vector Machine (SVM), KNN, and Random Forest (RF) has been employed for plant disease detection. For instance, the k-FLBPCM (filtered local binary pattern masks) with a contour-based segmentation method was proposed in (Le et al., 2020), where the extracted features were used to train an SVM classifier. Despite its strength on clean samples, the model fails when exposed to geometric distortion and complex backgrounds from field scenarios. Directional Local Quinary Patterns (DLQP) were used with the SVM in (Ahmad et al., 2020), and attained reasonable accuracy in some classes, but failed to perform well on leaves with noisy or cluttered areas. Another framework suggested in (Sun et al., 2019) combined SLIC clustering, Harris detector, and GLCM features to train an SVM to identify diseased areas in tea leaves. This approach achieved better accuracy but at the expense of increased computational complexity. Other image analysis methods include (Pantazi et al., 2019), who applied GrabCut to HSV transformations with LBP features at 75% accuracy, and (Kuricheti and Supriya, 2019), who applied GLCM and K-means clustering to classify turmeric leaves; however, the method was sensitive to light variations and unable to be used in real-time. In (Ramesh and Hebbar, 2018), Histogram of Oriented Gradients (HOG) and Random Forest were used to offer an efficient solution, but with reduced performance in high intra-class variance cases. These techniques have a dependency on domain knowledge while formulating handcrafted features, which often makes them biased toward specific, prominent symptoms while overlooking subtle indicators of the disease. The heavy reliance on image processing stages, including segmentation or color normalization, further complicates pipelines. These ML-based approaches require feature engineering and are not very scalable. As highlighted in (Shoaib et al., 2023), their performance becomes highly ineffective in large-scale or more challenging contexts, which encourages the adoption of DL-based approaches.

The application of state-of-the-art (SOTA) DL models has significantly improved the performance of plant disease detection over traditional approaches. Early works include (Argüeso et al., 2020), which used InceptionV3 and SVM in a few-shot learning model, and obtained 91.8% accuracy on a custom subset of the PlantVillage dataset, but with limited scalability. A CNN-based tomato disease classifier was suggested in (Agarwal et al., 2020) with 90.2% accuracy on the PlantVillage tomato subset, but with a high risk of overfitting due to low diversity. Richey et al (Richey et al., 2020). also trained ResNet50 on ImageNet and achieved 95% accuracy on real-field images of maize, though its high computational rate prevented its use on mobile devices. Zhang et al (Zhang et al., 2020). enhanced tomato disease identification with a Faster R-CNN pipeline, a ResNet backbone, and K-means clustering on the PlantVillage and PlantDoc data, with several symptoms being successfully categorized in a more complex situation. Batool et al (Ahmed and Yadav, 2023). combined AlexNet with KNN for binary tomato leaf classification on PlantVillage, providing early detection with a reasonable computation requirement, but rendering KNN inapplicable in real-time due to its high memory requirement. As DL matured, CNN architecture became more sophisticated. Architectures, such as VGG16, ResNet, and DenseNet, performed well. ResNet with CBAM attention achieved 99.9% accuracy on PlantVillage subset (Mohanty et al., 2016) for rice disease detection, and 98.6% accuracy on tomato-specific datasets (Zhang et al., 2023). DenseNet, with its dense connectivity, enhanced gradient flow, and reuse of features, achieved an accuracy of 99% on both PlantVillage and field datasets (Wang et al., 2025), but experienced overfitting in curated datasets. Segmentation-based models such as DeepLabV3 provided a pixel-wise severity analysis, although with substantial labeling effort (Geetharamani and Pandian, 2019). These models represent a significant advancement towards more complex image classification tasks, such as localization, severity assessments, and multi-disease classification. This development is crucial for field applications, where plants often exhibit overlapping symptoms that cannot be classified into a single disease category. At the same time, other lightweight convolutional neural networks like MobileNet, ShuffleNet, and EfficientNet allowed the use of neural networks on mobile and edge devices. MobileNet, which uses depth-wise separable convolution, reached 95% accuracy on maize and tomato data (Richter and Kim, 2025), the B0 EfficientNet model reached 73.3% accuracy on PlantDoc irrespective of background complexity (Devi et al., 2023). These lightweight models were sensitive to domain changes, although they were efficient.

Recently, transformer-based architectures such as Vision Transformers (ViT) and Swin Transformers have been used to classify plant diseases using global attention and refined spatial modeling (Ritharson et al., 2024). In some cases, ViT often performs better than traditional CNNs by avoiding convolutional inductive bias (Pacal and Işık, 2025), but it requires a large number of images for training and is computationally expensive, which limits its use in real-time. Pacal et al (Dolatabadian et al., 2025). showed that ViT and Swin Transformer models achieved comparable performance to CNN models on corn disease datasets, including ResNet and DenseNet. A hybrid ResNet and transformer architecture, ConvNeXt uses GELU activations and layer normalization (Wu et al., 2023). In recent times, a ViT-RoT model, using ConvNeXt-Small, resulted in 96.7% accuracy on tomato leaves, with improved generalization (Wang et al., 2023). Liu & Zhang introduced an Efficient Swin Transformer for plant disease detection, an improvement over the Swin-T architecture, optimized for lower computational cost while achieving a precision of 80.14% and a recall of 76.27% on the Plantdoc dataset (Liu and Zhang, 2025). Kalpana et al (Kalpana et al., 2024). proposed a novel ensemble of Swin transformers and residual networks integrated with feed-forward networks to improve hierarchical context extraction for complex leaf symptom patterns, achieving 99.95% accuracy. Furthermore, hybrid architectures have been proposed by combining the strengths of Convolutional Neural Networks (CNNs) and Vision Transformers (ViT), showing enhanced local-global feature fusion for multi-class disease recognition, achieving an accuracy rate of 99.24% and 98% for the apple and corn datasets (Aboelenin et al., 2025). Additional elements, such as attention fusion, have also led to an increase in performance, making hybrid CNN-transformer models a promising solution. Beyond accuracy, transformer-based models introduce an advantage in interpretability through attention maps, which allow visualization of regions in a leaf image contributing to a decision. This can help farmers or agronomists understand why a model is classifying a leaf as diseased, increasing trust in AI systems.

Most current state-of-the-art plant disease data, including PlantVillage, is recorded in controlled environments with even lighting, orientation, and a plain background. However, PlantDoc and other field-based datasets aim to represent real-world variability, including non-uniform illumination, cluttered backgrounds, and natural occlusions. Although applicable in benchmarking, such datasets do not reflect the diversity of field images in the real world, which include irregular lighting, overlapping leaves, occlusions, and complicated natural backgrounds (Radenović et al., 2019). Wang et al (PlantVillage Dataset). emphasized that this gap must be minimized by means of domain adaptation and synthetic data generation. Data augmentation and GAN-based synthesis have also been explored to reduce overfitting, but large-scale, annotated field datasets remain limited. Field datasets also pose the challenge of class imbalance, where some diseases are over-represented while others have very few examples. This imbalance can lead models to be biased toward common diseases, overlooking rare but highly damaging ones. To solve this problem, researchers have started using few-shot learning, active learning, and semi-supervised approaches, but progress is still limited. To provide a clearer comparative understanding, **Table 1** consolidates the performance metrics of the DL-based models discussed in this section.

**Table 1:** Review of existing plant disease detection approaches.

Ref	Model	Architecture type	PlantVillage accuracy (%)	PlantDoc accuracy (%)	Limitations
(Argüeso et al., 2020)	Compact CNN	Lightweight CNN	85–90%	~50–60%	not robust under occlusions and noisy backgrounds.
(Agarwal et al., 2020)	LeNet (Tomato leaf detection)	Lightweight CNN	~95.0	–	Poor generalization in real-time settings and complex variations in disease symptoms.
(Richey et al., 2020)	ResNet-50 (Mobile Version)	CNN	~95.0	–	Limited mobile scalability; unsuitable for portable devices.
(Zhang et al., 2020)	Faster R-CNN	Object Detection CNN	~97–99%	60–70	needs bounding box annotations and suffers from slow inference.
(Batool et al., 2020)	AlexNet + KNN	CNN + ML Hybrid	~96.0	Not reported	high computational cost.
(Mohanty et al., 2016)	CNN Ensemble	Ensemble CNN	~99.0	~70.0	computationally complex and unsuitable for edge devices.
(Zhang et al., 2023)	ResNet-50 + CBAM	CNN + Attention	99.97	~69.5	computationally complex and memory-intensive.
(Wang et al., 2025)	DenseNet-121	CNN	99.31–99.35	55–58	model over-fitting on a curated dataset and high computational cost.
(Geetharamani and Pandian, 2019)	DeepLabV3	Semantic Segmentation CNN	98–99%	67–72%	needs dense pixel-level labels and heavy computation.
(Richter and Kim, 2025)	MobileNetV2	Lightweight CNN	97.98	47.56	accuracy degrades under domain shift and complex background.
(Devi et al., 2023)	EfficientNet-B0	Lightweight CNN	99.62	57–62	lack of domain generalization and noise sensitivity.
(Dolatabadian et al., 2025)	Swin Transformer	Transformer	99.10	77.54	dependency on large-scale labelled data and is computationally complex.
(Wang et al., 2023)	ViT-RoT / ConvNeXt-Small	Transformer-Hybrid	96.7	72.85	dependency on large-scale annotated data and computationally complex.

This review of literature highlights the obvious shift of handcrafted ML models to DL models, lightweight networks, and transformer networks for plant disease detection. However, there are still difficulties in practical implementation. Domain variability still causes major performance degradation when applied to field images with noisy, cluttered, or irregular lighting. Overfitting is still a concern where training data sets are small, hence minimizing cross-crop and cross-environment generalization. Besides, most SOTA architectures are computationally intensive, which limits real-time or edge deployment. Future directions must therefore address a multi-faceted problem where methods should not only achieve high accuracy but also be lightweight enough for edge devices, robust to field variability, and able to handle multiple diseases across different plant species. This balancing act will define the next phase of research in AI-driven agricultural disease detection.

## 3 Proposed methodology

**Figure 1:**
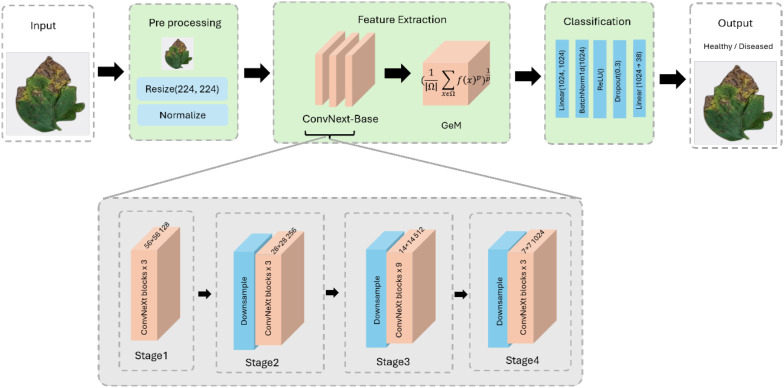
Architecture of the proposed ConvGeM-NeXt model, highlighting the ConvNeXt backbone, the integration of generalized mean (GeM) pooling, and the redesigned classification head used for plant leaf disease detection.

This section outlines the suggested methodology to develop a robust deep learning method for plant disease detection. We introduce different architectural enhancements in the ConvNeXt-Base architecture, selected for its balance between representational power and computational efficiency. More precisely, three architectural refinements were introduced (Lewandowski et al., 2017): the replacement of the standard Global Average Pooling (GAP) with a trainable Generalized Mean pooling layer to improve spatial discriminability (Shoaib et al., 2023), the design of a custom classifier head that integrates normalization, activation, and dropout layers to enhance regularization and minimize overfitting and (Ahmed and Yadav, 2023) the adoption of the ReLU activation function in classification to maintain sparsity and computational efficiency while ensuring stable gradient propagation. These adjustments allow the model to perform better not only on controlled datasets like PlantVillage but also enhance the ability to adapt to more challenging and realistic datasets like PlantDoc. The architectural details of the proposed framework is shown in **Figure 1**.

### 3.1 Pre-processing

We process all input images through a standardized preprocessing pipeline. All images were resized to 224×224 pixels to align with the input dimensionality conventionally employed in convolutional and transformer-based vision models. Besides, pixel values are normalized based on the mean and standard deviation values obtained on ImageNet. This pre-processing simplifies weight transfer learning, which is initially trained on ImageNet and yields faster convergence during training the model on the plant-related dataset. The same preprocessing strategy is applied to both PlantVillage and PlantDoc, allowing a fair and consistent evaluation across controlled and field conditions. This study does not use data augmentation methods, such as random cropping, color jittering, or flipping, to achieve robustness, as opposed to many other studies that use extensive data augmentation. This decision retains the natural properties of the datasets, ensuring that any performance gain is caused by architectural refinements rather than artificial variability in the dataset.

### 3.2 Architectural framework

The proposed ConvGem-Next architecture is built on ConvNeXt-Base, a modern convolutional network that incorporates improvements based on the transformer architecture design, but with the same efficiency as the residual-style convolutional architecture. The details are presented in subsequent sections.

#### 3.2.1 ConvNeXt backbone

ConvNeXt-Base serves as a strong base for feature extraction that can capture hierarchical spatial representations while keeping computational stability during training. This architecture was chosen as the backbone because it incorporates the inductive bias of convolutional networks and enhances them with transformer-based models, which represents a balance between efficiency and representation capacity. The ConvNeXt-Base model has 36 convolutional blocks arranged in four hierarchical stages, with a depth pattern of (Ahmed and Yadav, 2023; Ahmed and Yadav, 2023; Ahmed and Yadav, 2023; Zhang et al., 2023). As we move through these stages, the number of channels increases from 96 to 192, 384, and 768, such that we can capture increasingly abstract representations. The structure of each block is the same, with a two-dimensional LayerNorm, depthwise convolution with a 7×7 kernel, GELU activation, pointwise convolution for expansion, and stochastic depth regularization. While training, residual skip connections are added to increase the gradient stability. To achieve protocol consistency, ImageNet-1K pretrained weights were pre-initialized on all the layers and then fine-tuned to the plant disease datasets. This initialization enables transfer learning, faster convergence, and optimization, as well as permitting previously learned information obtained in high-scale visual spaces. Empirically, this backbone architecture preserved high-resolution spatial lesion patterns and improved convergence stability, particularly in high intra-class similarity, a common problem in plant pathology datasets.

#### 3.2.2 Generalized mean pooling

The second stage of the proposed model is the pooling mechanism, whose aim is to replace conventional uniform pooling with a more discriminative operation that can extract localized spatial patterns relevant to disease diagnosis. In many leaf disease images, the lesions or discolorations are localized to small regions of the leaf, and conventional global average pooling cannot emphasize such discriminative locations. To address this issue, Generalized Mean Pooling is used in place of GAP. GMP adds a learnable pooling exponent (ρ) that allows the model to interpolate between smooth average pooling (ρ = 1) and max pooling (ρ → ∞). GMP pooling is computed as shown in Equation 1.

(1) $$f_{GMP}=\;{\left(\frac{1}{\left|\text{Ω}\right|}{\sum_{x\epsilon \text{Ω}}f\left(x\right)}^{p}\right)}^{\frac{1}{p}}$$

Where f(x) represents the activation at the spatial location x, Ω is the set of all spatial locations, and ρ is the learnable parameter initialized at 3. During training, the network dynamically adapts the value of ρ, enabling it to selectively put the most informative spatial areas in the foreground but still retain global feature map coverage. This adaptive pooling method ensures the network preserves high-resolution lesion information and increases spatial discriminability. Therefore, GMP significantly improves the model’s sensitivity to low-intensity visual clues, particularly in datasets with natural variations such as uneven illumination or partial occlusions. Unlike GAP, which smooths representations uniformly, GMP emphasizes subtle texture differences and localized lesion features, improving the model’s ability to generalize to in-field scenarios with weak or partially occluded symptoms. Previous studies in fine-grained recognition suggest that GMP may enhance recall for rare classes by better preserving discriminative local details, highlighting its potential importance for agricultural applications (Radenović et al., 2019).

#### 3.2.3 Custom classification head

The classification head was also restructured to improve regularization and feature separability. The native ConvNeXt-Base classifier utilizes only one linear projection, which is usually not adequate for the case of images of plant diseases containing highly intra-class similar and cross-domain variable datasets. In this, the 1024-dimensional feature vector aggregated from the backbone is first converted by a fully connected layer of the same size. Batch normalization follows immediately after that for normalizing the gradients and improving training convergence. Non-linearity is introduced by a ReLU activation, and a dropout layer with probability p=0.3 prevents overfitting by applying stochastic neuron deactivation during training. The feature vector is subsequently passed to the final linear layer that projects the representation to the target label space, which is 38 classes for PlantVillage and 27 classes for PlantDoc. This head adaptation contributes about 1.05 million trainable parameters, bringing the total number of parameters to about 89 million. Despite its significance, the computational expense remains negligible in comparison with the size of the backbone. More importantly, the customized head improves the discriminative power of the model, allowing for more uniform discrimination between visually similar disease classes and improving the generalizability of the model to new samples. Practically, this restructuring avoided memorization of small leaf patterns on PlantVillage and improved classification margins on PlantDoc, where domain shifts are high. The customized head thus ensures that embeddings learned remain compact and discriminative, allowing robust performance on controlled lab datasets as well as real-world field images.

### 3.3 Training setup and optimization strategy

Training was conducted on Google Cloud TPU v3–8 instances using the torch-xla framework for distributed optimization. The TPU configuration provides 8 cores with 128 GB high-bandwidth memory, supported by 64 GB of system RAM for data loading and preprocessing. No dedicated GPU was used, as TPU acceleration was sufficient for stable and efficient training of the ConvGeM-NeXt model on both PlantVillage and PlantDoc datasets. The AdamW optimizer in our model isolates the weight decay from gradient updates for the model to improve generalization. The learning rate was set to 1×10^-3^, with a weight decay of 1×10^−4^. In our work, we added a Cosine Annealing pattern as a learning rate scheduler with T_max_​=30 epochs, to achieve smooth convergence. Each training session is set to run for at least 30 epochs, with a batch size of 64. The model is trained using the standard cross-entropy loss function. All optimizer steps are executed with xm.optimizer_step() to ensure synchronization across TPU cores. The training wall time is approximately 10–12 minutes for one epoch on the PlantVillage dataset and slightly shorter for PlantDoc. The final reported results correspond to the model state obtained at the last training epoch. Validation loss was monitored to analyze convergence behavior and potential overfitting. To ensure stable and reliable performance evaluation, we repeated each experiment three times using different random seeds but keeping the identical data split, architectures, and hyperparameters. Performace is summarized using mean ± standard deviation and 95% confidence intervals for accuracy were calculated. **Table 2** summarizes the full training configuration, including input preprocessing, optimization parameters, training setup, and hardware used.

**Table 2:** Training configuration and implementation details for the ConvGeM-NeXt model.

Category	Parameter	Description
Input Pre-processing	Image resizing	224 × 224 pixels
	Image format	RGB
	Normalization	ImageNet mean and standard deviation
Optimization	Optimizer	AdamW
	Learning rate	1 × 10^−3^
	Weight decay	1 × 10^−4^
	Loss function	Cross-entropy
	Learning rate scheduler	Cosine Annealing
	Scheduler parameter	Tmax = 30 epochs
Training Setup	Batch size	64
	Number of epochs	30
	Model selection	Lowest validation loss
Hardware	Accelerator	Google Cloud TPU v3-8 (via torch-xla environment)
	Framework	PyTorch with torch-xla

## 4 Experiments and results

This section presents the experimental evaluation of the proposed ConvGeM-NeXt framework. The details of datasets and evaluation metrics adopted for performance measurement are also provided. The results are reported on both controlled and real-world datasets, with emphasis on assessing classification accuracy and robustness across diverse disease categories.

### 4.1 Datasets

This section presents the details of both the plant village and plant doc datasets employed to assess the competency of the proposed method. For the experimentation protocol, we employed the standard split of 70% for training, 15% for validation, and 15% for testing for both datasets. The comparison of both datasets are shown in **Table 3**.

**Table 3:** Comparison of PlantVillage and PlantDoc datasets.

Dataset	No. of images	No. of classes	Key characteristics
PlantVillage (PlantVillage Dataset)	>54,000	38	Clean background, uniform lighting, isolated leaves, minimal noise
PlantDoc (PlantDoc Dataset)	2,598	27	Complex background, illumination variation, occlusion, intra-class variability

#### 4.1.1 PlantVillage: controlled dataset

**Figure 2:**
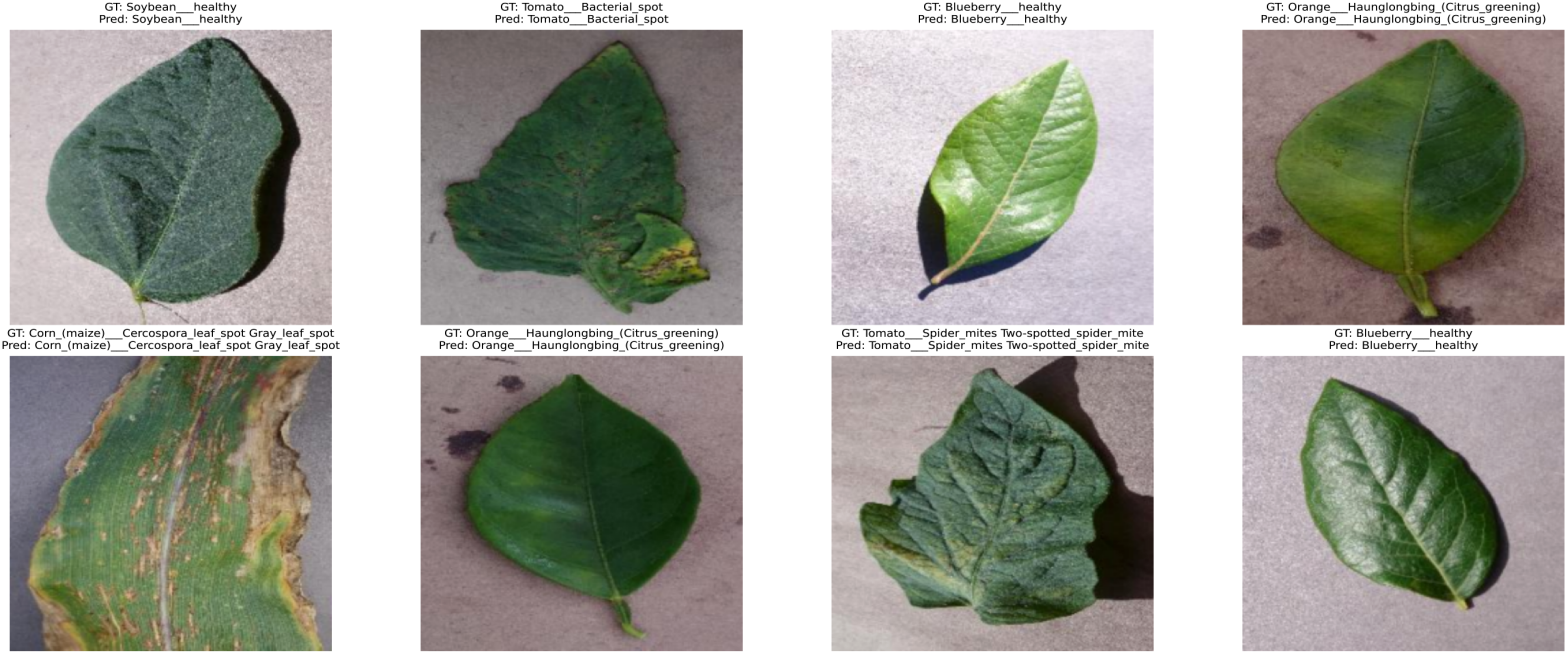
Representative sample images from the PlantVillage dataset, illustrating leaf disease categories captured under controlled imaging conditions with uniform backgrounds and lighting.

PlantVillage (PlantVillage Dataset) is a freely accessible, large-scale image database that is used to provide a standardized benchmark on the performance of deep learning methods in plant disease recognition. It consists of over 54000 high-resolution RGB images of crop leaves, spread in 38 categories, including both healthy and diseased leaves of various crops, including tomato, apple, corn, grape, potato, and pepper. All of the images are matched to a certain type of plant species and disease condition, which allows a wide and equal representation of the agricultural pathology under the controlled environment. The data were filtered in very high standards of laboratory conditions, with clean, plain backgrounds, fixed light, and placement of leaves. These conditions reduce the effects of environmental artifacts, including shadowing, occlusion, and background interference, to ensure the learning process is only about disease-related visual images. All the images included in the dataset were taken at a high resolution and resized to 224×224 pixels as per model requirements. This dataset has other specific benefits in terms of its clean, balanced, and artifact-free composition, and is especially useful in training and fine-tuning deep learning models. In addition, the class distribution in the dataset is also fair in representing different crop-disease combinations, which in training balances the gradient updates and in the convergence behavior of the model. To demonstrate the visual attributes of the PlantVillage data, **Figure 2** shows some sample images of the leaves of various crop diseases. The images highlight the controlled acquisition environment with uniform backgrounds and lighting, ensuring that disease features are the primary visual cues. Furthermore, **Table 4** provides a detailed breakdown of the number of samples per class, demonstrating the dataset’s balanced representation of healthy and diseased leaves across the 38 categories.

**Table 4:** Class-wise distribution of the PlantVillage dataset.

Class name	Total samples
Apple:_Apple_scab	630
Apple:_Black_rot	621
Apple:_Cedar_apple_rust	275
Apple:_healthy	1,638
Blueberry:_healthy	1,502
Cherry_(including_sour):_Powdery_mildew	1,052
Cherry_(including_sour):_healthy	854
Corn_(maize):_Cercospora_leaf_spot Gray_leaf_spot	513
Corn_(maize):Common_rust	1,192
Corn_(maize):_Northern_Leaf_Blight	985
Corn_(maize):_healthy	1,162
Grape:_Black_rot	1,180
Grape:_Esca_(Black_Measles)	1,383
Grape:_Leaf_blight_(Isariopsis_Leaf_Spot)	1,076
Grape:_healthy	423
Orange:_Haunglongbing_(Citrus_greening)	5,507
Peach:_Bacterial_spot	2,297
Peach:_healthy	360
Pepper,_bell:_Bacterial_spot	997
Pepper,_bell:_healthy	1,478
Potato:_Early_blight	1,000
Potato:_Late_blight	1,000
Potato:_healthy	152
Raspberry:_healthy	371
Soybean:_healthy	5,090
Squash:_Powdery_mildew	1,835
Strawberry:_Leaf_scorch	1,109
Strawberry:_healthy	456
Tomato:_Bacterial_spot	2,127
Tomato:_Early_blight	1,000
Tomato:_Late_blight	1,901
Tomato:_Leaf_Mold	952
Tomato:_Septoria_leaf_spot	1,771
Tomato:_Spider_mites Two-spotted_spider_mite	1,676
Tomato:_Target_Spot	1,404
Tomato:_Tomato_Yellow_Leaf_Curl_Virus	5,357
Tomato:_Tomato_mosaic_virus	373
Tomato:_healthy	1,585
Total Images	54,284

#### 4.1.2 PlantDoc: real-world evaluation corpus

**Figure 3:**
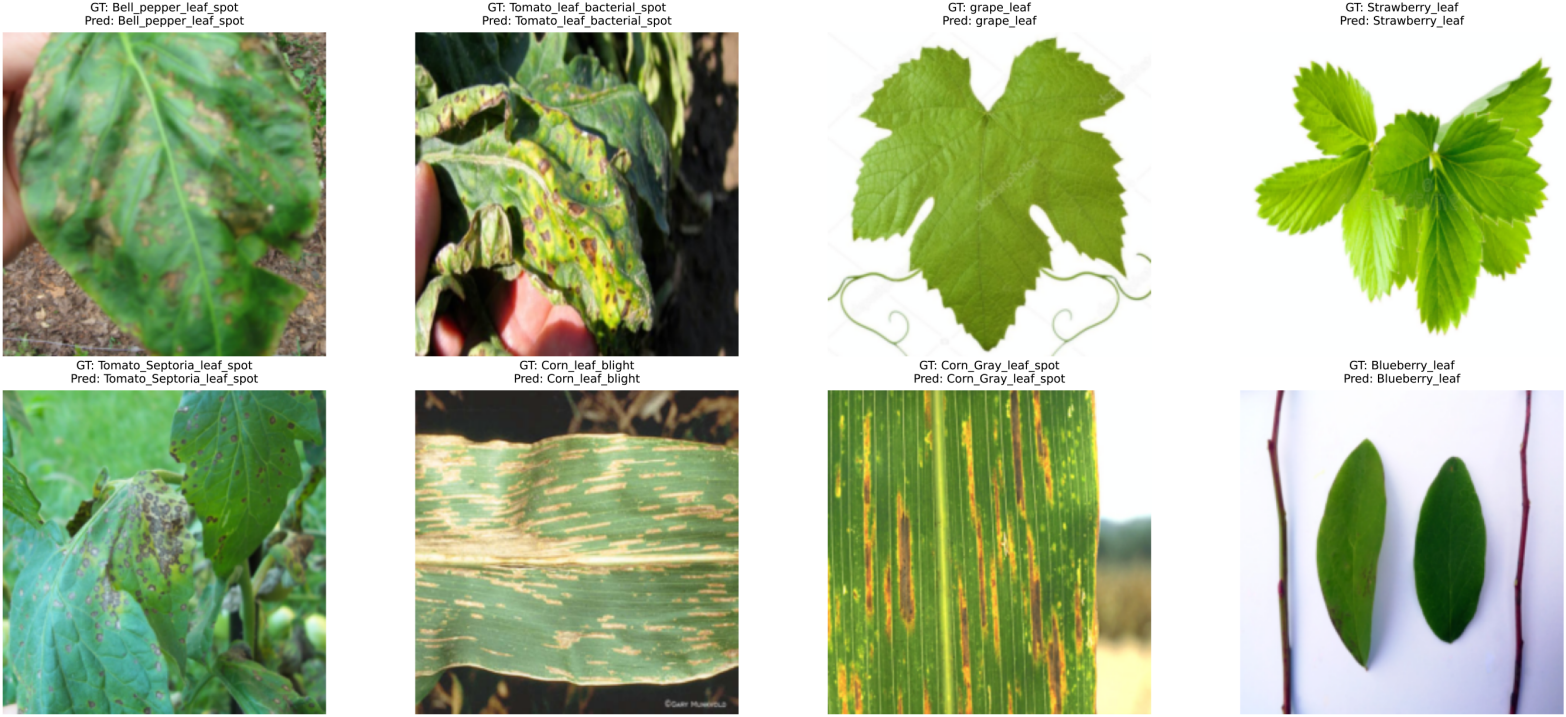
Representative sample images from the PlantDoc dataset, demonstrating real-world leaf disease samples acquired under diverse field conditions with varying backgrounds, illumination, and occlusions.

The PlantDoc dataset (PlantDoc Dataset) comprises data collected from the field and includes 2,598 RGB images that are labeled across 27 different classes of plant diseases, as shown in **Figure 3**. The images are captured in a real-world setting, with natural backgrounds, varying lighting conditions, leaf obstructions, overlapping plant structures, and considerable intra-class variability. Such characteristics make the dataset ideal for evaluating how well a model performs outside the controlled lab environment. In contrast to the clear and well-defined symptoms of the diseases in the case of PlantVillage, which are observed in the laboratory environment, the images in PlantDoc often contain blurred boundaries and noisy visual cues, reflecting the challenges faced in real-world scenarios. The model’s performance on the PlantDoc dataset serves as a crucial benchmark for assessing its robustness under real-world, uncontrolled environmental conditions. To show the visual attributes of the PlantDoc dataset, **Figure 3** presents representative sample images from different plant species and disease categories, highlighting the natural variability in lighting, backgrounds, leaf orientations, and occlusions. The real-world, uncontrolled conditions under which PlantDoc images are captured make it an effective benchmark for evaluating the robustness of plant disease recognition models outside controlled laboratory settings. **Table 5** provides the number of samples per class, illustrating the dataset’s distribution across the 27 categories.

**Table 5:** Class-wise distribution of the PlantDoc dataset.

Class name	Total samples
Apple_Scab_Leaf	93
Apple_leaf	88
Apple_rust_leaf	106
Bell_pepper_leaf	42
Bell_pepper_leaf_spot	83
Blueberry_leaf	117
Cherry_leaf	57
Corn_Gray_leaf_spot	67
Corn_leaf_blight	194
Corn_rust_leaf	117
Peach_leaf	112
Potato_leaf_early_blight	171
Potato_leaf_late_blight	208
Raspberry_leaf	119
Soyabean_leaf	65
Squash_Powdery_mildew_leaf	130
Strawberry_leaf	96
Tomato_Early_blight_leaf	88
Tomato_Septoria_leaf_spot	157
Tomato_leaf	52
Tomato_leaf_bacterial_spot	110
Tomato_leaf_late_blight	111
Tomato_leaf_mosaic_virus	54
Tomato_leaf_yellow_virus	238
Tomato_mold_leaf	91
Tomato_two_spotted_spider_mites_leaf	2
grape_leaf	75
grape_leaf_black_rot	79
Total Images	2,922

### 4.2 Evaluation metrics

To assess the model’s performance, we used Top-1 accuracy, macro-averaged precision, and macro-averaged F1-score. In addition, Mean Class Accuracy (MCA), also referred to as Mean Average Accuracy, is used to quantify the average classification accuracy across all disease classes. These metrics work well together because they show both overall accuracy and class-balanced performance, which is important in plant pathology datasets where classes are not evenly distributed. Top-1 accuracy is the number of samples predicted correctly divided by the total number of samples, as defined in Equation 2.

(2) $$Accuracy=\;\frac{Number\;of\;Correct\;Predictions}{Number\;of\;Total\;Samples}$$

Mean Class Accuracy is defined as the arithmetic mean of per class accuracies and is computed as shown in Equation 3.

(3) $$Mean\;Class\;Accuracy=\;\frac{1}{C}\sum_{i=1}^{C}\frac{TP_{i}}{N_{i}}$$

where TP_i_ represents the correctly classified samples of class *i* and N_i_ denotes the total number of samples belonging to class *i*.

While accuracy is a direct measure of correctness, it is deceptive in imbalanced datasets as it is significantly biased towards majority classes. Macro-averaged measures are employed for this limitation. Macro precision and macro recall are computed as follow in Equations 4, 5, respectively.

(4) $$Macro\;Precision=\;\frac{1}{C}\sum_{i=1}^{C}\frac{TP_{i}}{TP_{i}+\;FP_{i}}$$

(5) $$Macro\;Recall=\;\frac{1}{C}\sum_{i=1}^{C}\frac{TP_{i}}{TP_{i}+\;FN_{i}}$$

Where *C* is the number of classes, $FP_{i}$ and $FN_{i}$ denotes the false positives and false negatives, respectively.Each class is assigned equal weight regardless of its sample size, ensuring that minority and rare disease classes contribute equally to the overall evaluation and reducing bias toward majority classes. Unlike Top-1 accuracy, Mean Class Accuracy ensures that each disease category contributes equally to the final score, making it particularly suitable for evaluating robustness under class imbalance.

Macro F1-score is the harmonic mean of macro precision and macro recall, as shown in Equation 6. This metric is interested in the false positive vs. false negative trade-off and is thus of particular significance in plant disease classification tasks where premature misclassification of latent or rare diseases has serious implications for agriculture.

(6) $$Macro\;F1=\frac{2\times \;Macro\;Recall\;\times \;Macro\;Precision}{Macro\;Recall+\;Macro\;Precision}$$

All the measures are implemented using the scikit-learn library, and validation set and test set estimations are conducted to capture both in-sample learning as well as generalization to out-of-sample cases. Although Top-1 accuracy provides a high-level estimate of accuracy, it fails to capture fairness across diverse classes. Macro metrics avoid such a limitation by capturing deficiencies in under-represented disease classes, and Macro F1-score, in return, provides a measure of model robustness in balancing sensitivity and specificity under real-world field conditions where visual cues can be weak, noisy, or occluded. These measures provide a better estimation of model generalization, fairness, and robustness across controlled and in-field environments.

### 4.3 Quantitative evaluation

**Figure 4:**
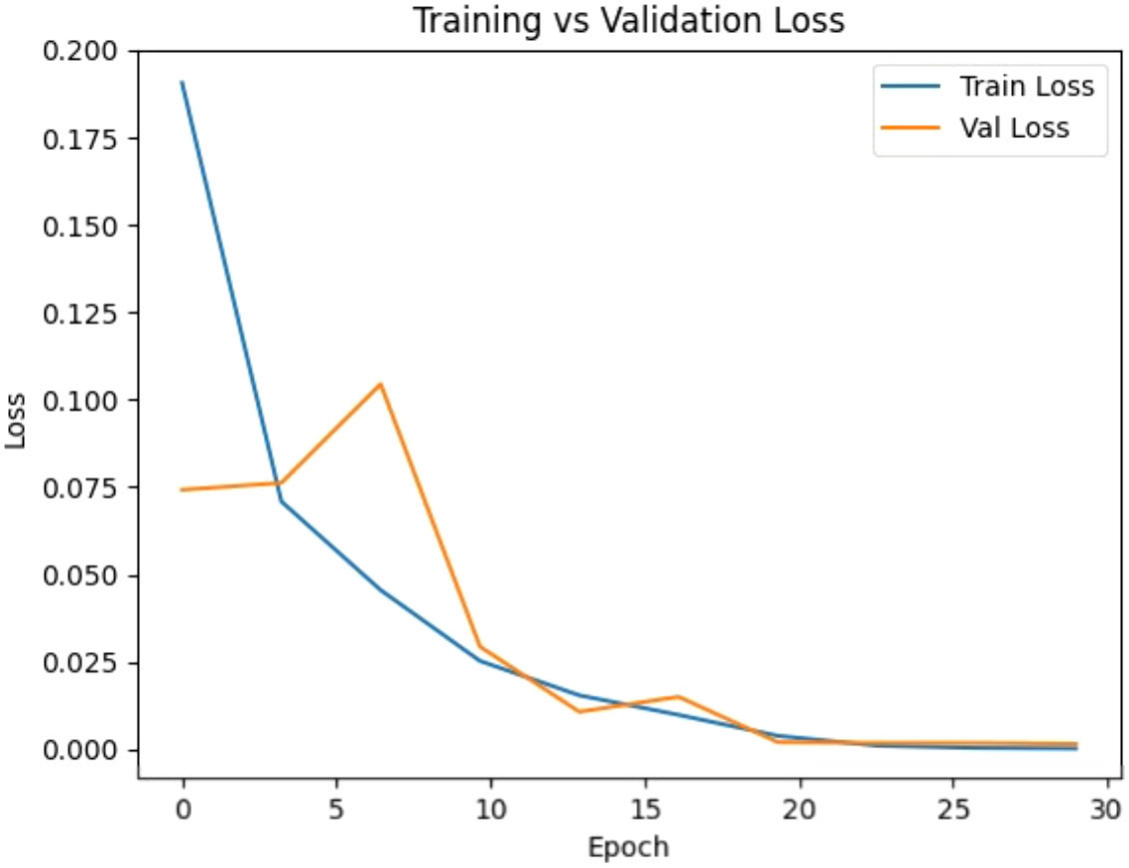
Training and validation accuracy and loss curves of the proposed ConvGeM-NeXt model on the PlantVillage dataset, illustrating convergence behavior and generalization performance across epochs.

**Figure 5:**
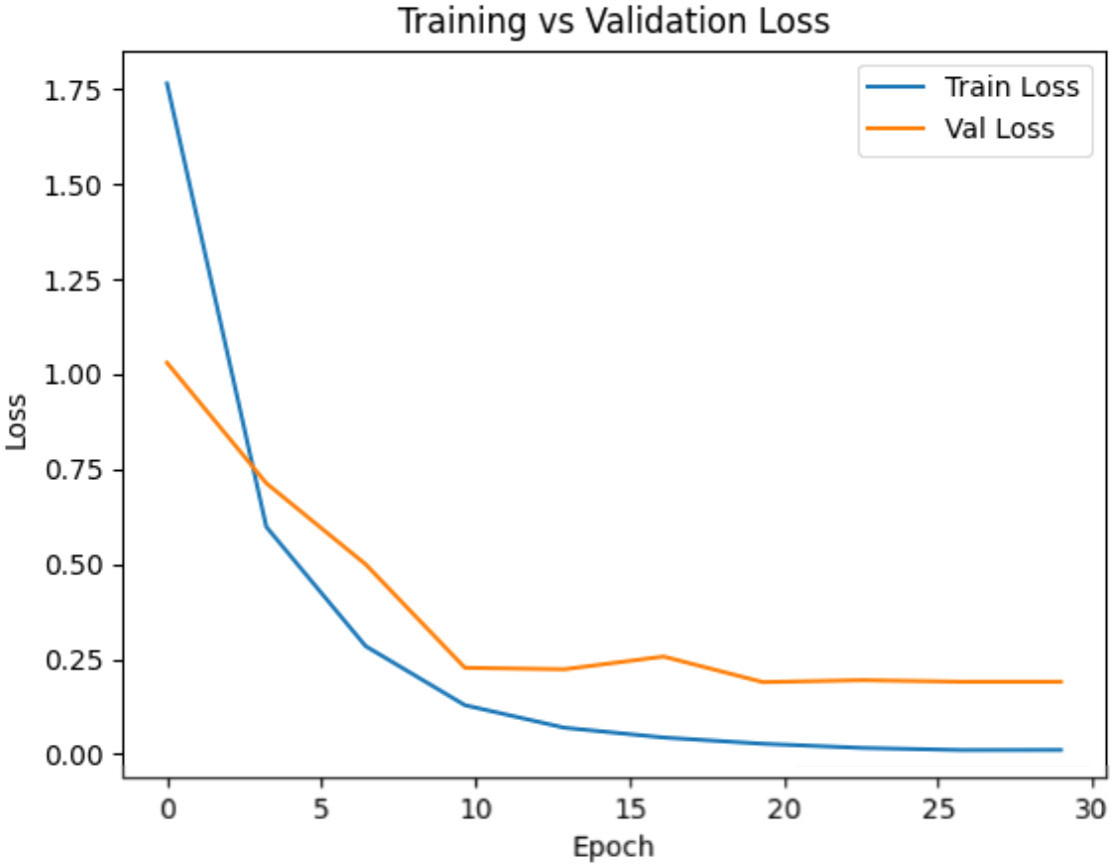
Training and validation accuracy and loss curves of the proposed ConvGeM-NeXt model on the PlantDoc dataset, illustrating convergence behavior and generalization performance across epochs.

The objective of this evaluation is to assess the ability of the proposed model to generalize across curated datasets and real-world conditions. The performance was tested on PlantVillage and PlantDoc in controlled and real-world environments based on Top-1 accuracy, macro precision, and macro F1-score. Accuracy is the overall accuracy of the model, and macro-averaged measures give equal weightage to all classes, thus giving sensitivity to rare or visually indistinguishable diseases. On PlantVillage, the proposed method performed extremely well with a Top-1 accuracy of 99.65% and a macro F1-score of 0.9959. These results demonstrate that it can detect extremely fine distinctions among 38 classes, including low-representation ones. Additionally, when the model was evaluated on the more challenging PlantDoc dataset of 27 classes collected under varied field conditions, it also performed well, with an accuracy of 94.69%, a Macro Precision of 0.9477, and a Macro F1-score of 0.9465 (**Table 6**). To evaluate the stability of performance under stochastic training conditions, each of the experiments was repeated three times with varying random seeds, with the same data splits and hyperparameters. ConvGeM-NeXt was able to perform significantly well with 99.73 ± 0.24% accuracy and CI 99.73 ± 0.27 on PlantVillage with very stable performance (**Table 7**). The model was also found to have a higher model accuracy of 92.19 ± 2.58 and a CI of 92.19 ± 2.92 when used across domains, on PlantDoc. Compared to baseline ConvNeXt-Base, our ConvGeM-NeXT method achieved better results on both datasets. For PlantVillage, the baseline had 96.19% accuracy, while the proposed method enhanced this to 99.65%. For PlantDoc, baseline attained 91.84% accuracy and 0.9231 Macro F1-score, while the proposed model achieved improved performance with 94.69% accuracy and 0.9465 Macro F1-score. To further analyze model robustness and potential overfitting behavior, we evaluated the training-validation divergence and generalization gap on both datasets using the learning curves shown in **Figures 4** and **5**. These findings reveal that the architectural refinements involving the GMP and ReLU activation in the reorganized classification head combined improve the performance of our method for plant disease detection. While the improvements are modest in controlled settings, they become significant in noisy real-world environments, confirming the model’s reliability for practical plant disease diagnosis.

**Table 6:** Performance comparison of the proposed model on the PlantVillage and PlantDoc datasets.

Dataset	Accuracy (%)	Test loss	Macro precision	Macro F1 score
PlantVillage	99.65	0.0133	0.99	0.99
PlantDoc	94.69	0.2552	0.95	0.95

**Table 7:** Cross-run accuracy, macro-precision, and macro-F1 performance of the proposed model on the PlantVillage and PlantDoc datasets.

Dataset	Accuracy (%)	Macro precision	Macro F1 score
PlantVillage	99.73 ± 0.24	0.992 ± 0.008	0.992 ± 0.008
PlantDoc	92.19 ± 2.58	0.923 ± 0.025	0.923 ± 0.025

### 4.4 Class-wise performance analysis

The goal of this experiment was to assess how well the model performs on each disease class, highlighting strengths and weaknesses in its predictions. This class-wise evaluation provides insights into the model’s robustness, helps identify underperforming classes, and guides future improvements for balanced disease detection performance.

#### 4.4.1 Performance on PlantDoc

This experiment was aimed at measuring the competency of the proposed ConvGem-NeXt model on the challenging PlantDoc dataset. Unlike curated datasets, PlantDoc reflects the complexity of real-world agricultural conditions, encompassing class imbalance, environmental noise, and considerable intra-class variability across 27 disease categories. This renders PlantDoc as a potential dataset for evaluating the generalization ability of the model in circumstances that one would anticipate upon implementation. The analysis involved accuracy, recall, and the F1-score of each category. To account for the imbalance in the number of samples in categories, a macro-averaged F1-score was calculated to evaluate the overall performance across all classes.

As demonstrated in **Table 8**, the model consistently produces better results. The 94.7% macro-averaged F1 score shows balanced classification performance across all classes, even under noisy and variable conditions. In addition, our method achieved an MCA of 87.04% on the PlantDoc dataset, demonstrating reliable performance across all disease categories despite class imbalance and challenging real-world conditions. Class-wise analysis demonstrated that precision ranged from 1.00 for Soybean Leaf to 0.50 for Potato Leaf Early Blight and Apple Leaf. The trend of recall values was similar across the classes, with 0.50 for the Potato Leaf Early Blight, 1.00 for the Tomato Leaf Late Blight, and 1.00 for Cherry Leaf and Peach Leaf. Our method reported the lowest recall score of 0.50 for the Potato Leaf Early Blight class. This is attributed to the strong resemblance of Potato Leaf Early Blight leaves to other classes, high variability under field conditions, and noisy and limited samples. These conditions make it harder for the model to extract useful features and accurately identify the disease. Collectively, the results of this experiment imply that our method works well in classifying many diverse classes and even distinguishes among classes having similar disease and leaf patterns with better accuracy. These results demonstrate that the architectural innovations in our ConvGeM-NeXt model show considerable strength and adaptability to field data, although distinguishing between visually very similar diseases still needs improvement.

**Table 8:** Class-wise performance of proposed models on the PlantDoc dataset.

Class name	Precision (%)	Recall (%)	F1-score (%)
Apple_Scab_Leaf	100.00	100.00	100.00
Apple_leaf	50.00	100.00	66.67
Apple_rust_leaf	100.00	100.00	100.00
Bell_pepper_leaf	100.00	100.00	100.00
Bell_pepper_leaf_spot	100.00	100.00	100.00
Blueberry_leaf	100.00	100.00	100.00
Cherry_leaf	100.00	100.00	100.00
Corn_Gray_leaf_spot	100.00	100.00	100.00
Corn_leaf_blight	100.00	100.00	100.00
Corn_rust_leaf	100.00	100.00	100.00
Peach_leaf	100.00	100.00	100.00
Potato_leaf_early_blight	50.00	50.00	50.00
Potato_leaf_late_blight	0.00	0.00	0.00
Raspberry_leaf	100.00	100.00	100.00
Soyabean_leaf	100.00	100.00	100.00
Squash_Powdery_mildew_leaf	100.00	100.00	100.00
Strawberry_leaf	100.00	100.00	100.00
Tomato_Early_blight_leaf	50.00	100.00	66.67
Tomato_Septoria_leaf_spot	100.00	100.00	100.00
Tomato_leaf	100.00	100.00	100.00
Tomato_leaf_bacterial_spot	100.00	100.00	100.00
Tomato_leaf_late_blight	100.00	100.00	100.00
Tomato_leaf_mosaic_virus	0.00	0.00	0.00
Tomato_leaf_yellow_virus	0.00	0.00	0.00
Tomato_mold_leaf	33.33	100.00	50.00
grape_leaf	100.00	100.00	100.00
grape_leaf_black_rot	100.00	100.00	100.00

#### 4.4.2 Performance on PlantVillage

This experiment was designed to assess the performance of our method on the PlantVillage dataset, comprising 38 diverse groups of healthy and diseased leaf samples. For each class, precision, recall, and F1-score were computed, while macro- and weighted-average scores were calculated to assess the overall classification performance. This assessment serves as a critical baseline for use in comparing performance between field and laboratory conditions.

These results (**Table 9**) confirm that the model had nearly perfect classification on all classes of the PlantVillage dataset. Out of 38 categories, over 90% of categories had a perfect precision, recall, and F1-score of 1.00. The overall macro-averaged F1-score was 0.99 with a weighted average of 1.00, demonstrating that almost all cases were classified correctly. The proposed method attained an MCA of 98.81% on the PlantVillage dataset, revealing that remarkable accuracy is consistently achieved across all 38 disease classes. Notably, diseases such as Apple Scab, Grape Black Rot, Orange Huanglongbing, and Tomato Yellow Leaf Curl Virus achieved flawless results, underscoring our model’s ability to extract discriminative features strongly for many diverse leaf classes. Slightly lower scores were observed for classes such as Tomato Early Blight (F1 = 0.96), Corn Cercospora Leaf Spot, and Potato Healthy (~0.93-0.95). These deviations suggest that intra-class variation and overlapping symptoms may still challenge classification accuracy, even in controlled environments. The obtained results clearly indicate that ConvGeM-Next has achieved superior classification performance on the PlantVillage dataset samples captured in controlled settings.

**Table 9:** Class-wise performance of proposed models on the PlantVillage dataset.

Class name	Precision (%)	Recall (%)	F1-score (%)
Apple:_Apple_scab	100.00	98.41	99.20
Apple:_Black_rot	100.00	98.41	99.20
Apple:_Cedar_apple_rust	100.00	100.00	100.00
Apple:_healthy	97.59	98.78	98.18
Blueberry:_healthy	99.34	100.00	99.67
Cherry_(including_sour):_Powdery_mildew	100.00	100.00	100.00
Cherry_(including_sour):_healthy	98.85	100.00	99.42
Corn_(maize):_Cercospora_leaf_spot Gray_leaf_spot	85.00	98.08	91.07
Corn_(maize):*Common_rust*	99.17	100.00	99.59
Corn_(maize):_Northern_Leaf_Blight	98.89	89.90	94.18
Corn_(maize):_healthy	99.15	100.00	99.57
Grape:_Black_rot	99.16	100.00	99.58
Grape:_Esca_(Black_Measles)	100.00	99.28	99.64
Grape:_Leaf_blight_(Isariopsis_Leaf_Spot)	100.00	100.00	100.00
Grape:_healthy	100.00	100.00	100.00
Orange:_Haunglongbing_(Citrus_greening)	100.00	99.64	99.82
Peach:_Bacterial_spot	100.00	100.00	100.00
Peach:_healthy	100.00	100.00	100.00
Pepper,_bell:_Bacterial_spot	100.00	100.00	100.00
Pepper,_bell:_healthy	100.00	99.32	99.66
Potato:_Early_blight	99.01	100.00	99.50
Potato:_Late_blight	96.15	100.00	98.04
Potato:_healthy	100.00	87.50	93.33
Raspberry:_healthy	100.00	100.00	100.00
Soybean:_healthy	99.61	100.00	99.80
Squash:_Powdery_mildew	100.00	100.00	100.00
Strawberry:_Leaf_scorch	100.00	100.00	100.00
Strawberry:_healthy	100.00	100.00	100.00
Tomato:_Bacterial_spot	99.06	98.59	98.82
Tomato:_Early_blight	95.19	99.00	97.06
Tomato:_Late_blight	98.92	96.34	97.61
Tomato:_Leaf_Mold	96.97	100.00	98.46
Tomato:_Septoria_leaf_spot	100.00	94.94	97.41
Tomato:_Spider_mites Two-spotted_spider_mite	98.25	100.00	99.12
Tomato:_Target_Spot	97.84	96.45	97.14
Tomato:_Tomato_Yellow_Leaf_Curl_Virus	99.81	100.00	99.91
Tomato:_Tomato_mosaic_virus	100.00	100.00	100.00
Tomato:_healthy	99.38	100.00	99.69

### 4.5 Confusion matrix analysis

**Figure 6:**
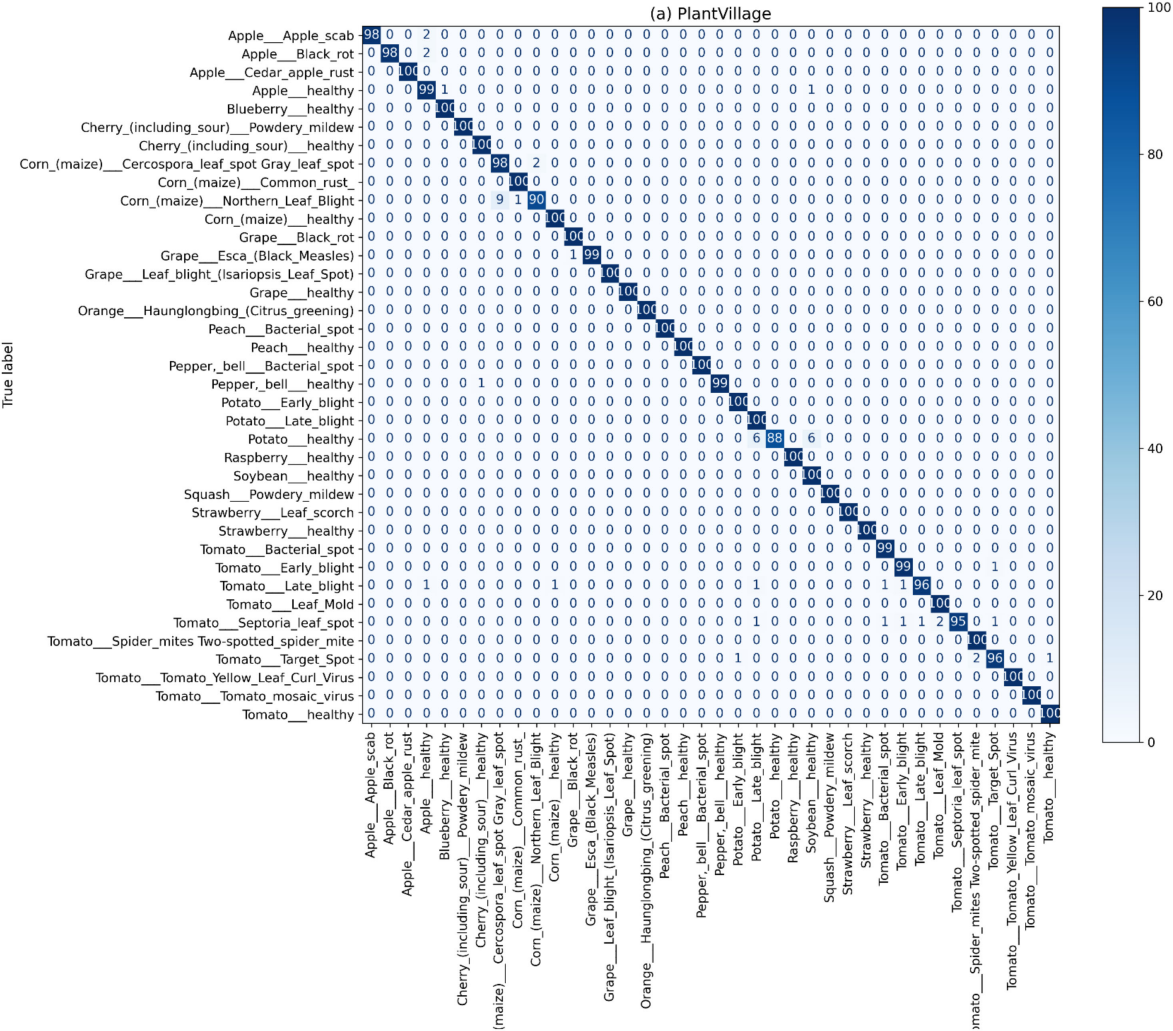
Normalized confusion matrix for the PlantVillage dataset, where strong diagonal dominance indicates high class-wise accuracy across 38 disease categories, and minor off-diagonal entries highlight limited misclassification among visually similar diseases.

The objective of this experiment was to assess the class-wise prediction performance in terms of identifying the false positives and false negatives on controlled PlantVillage and real-world PlantDoc datasets. Normalized confusion matrices were created to understand per-class accuracy, as well as the patterns of misclassification across all the disease types. Normalized confusion matrices presented in **Figure 6** show the proposed model’s class-wise prediction outcomes. The model accuracy, as demonstrated by the confusion matrices, shows dominant diagonals and, thus, correct classifications of the majority of the samples with only a few misclassifications. The main misclassifications occur with the classes “Tomato Early Blight” and “Tomato Septoria Leaf Spot” that have similar visual characteristics. The overlapping visual characteristics and similar symptoms of the class contain the diagnosed symptoms in the confusion patterns. The misclassification patterns do not significantly compromise the overall performance, indicating that the model can reliably recognize most disease classes.

**Figure 7:**
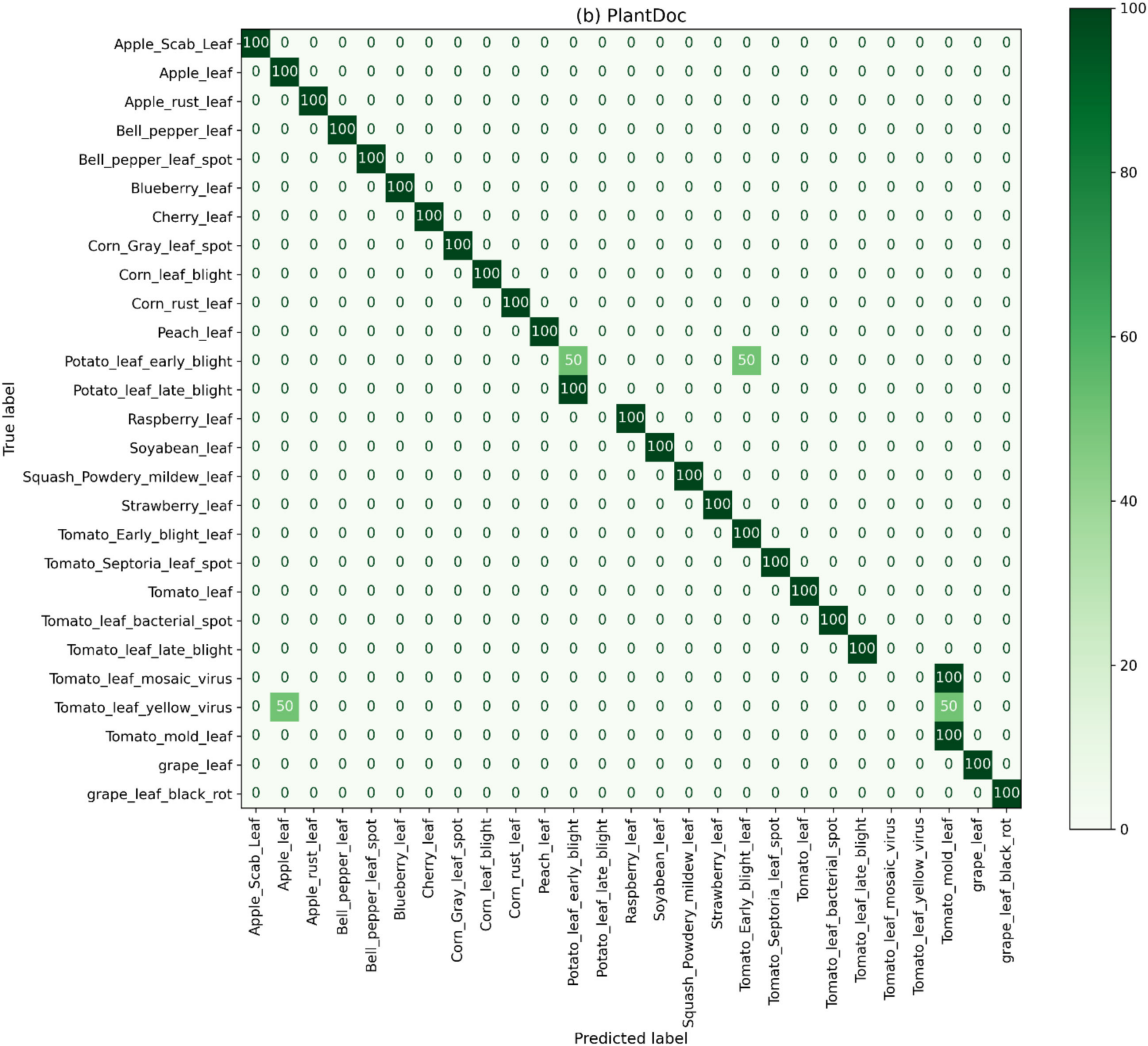
Normalized confusion matrix for the PlantDoc dataset, illustrating classification performance across 27 real-world disease classes, with diagonal dominance reflecting robust generalization and clustered misclassifications revealing challenges due to background clutter and symptom similarity.

The confusion matrix analysis for the PlantDoc corpus is depicted in **Figure 7**. The matrix shows that the model is highly accurate and consistent in the majority of disease classes, which proves the ability of our method for better generalizability. We observed some misclassification trends among morphologically related classes of disease, especially in the Potato and Tomato leaf groups. The confusion matrix shows false positives and false negatives of Potato Leaf Early Blight and Potato Leaf Late Blight, as the lesion structures and texture patterns are similar to each other. Likewise, Tomato Leaf Mosaic Virus, Tomato Leaf Yellow Virus, and Tomato Mold Leaf had several false predictions due to similarity in symptoms and color distortions. In Apple Leaf and Tomato Early Blight Leaf, minor false positives were also observed, where all other visually related samples were wrongly classified. Despite these localized confusions, the matrix shows great diagonal dominance, which means that the suggested ConvGeM-Next model can identify most of the plant disease classes even in cases of complex visual variations in the real-world field imagery.

### 4.6 Ablation study

This ablation study was done to examine the role of each architectural component within the ConvGeM-Next framework and to gain deeper insight into how each design decision influences the overall performance. The baseline ConvNeXt model, which was trained on the PlantVillage dataset, reached an accuracy of 96.19% and a test loss of 0.0609. We compared the performance of all modifications against this baseline. We systematically evaluated each modification by changing only one parameter at a time, such as the activation function, kernel size, block depth, pooling, or classifier head design, but all other parameters were held constant to assess the impact of each modification.

The outcome of this ablation study (**Table 10**) showed the impact of each architectural refinement. Activation function choice decisively affected performance, while ReLU maintained baseline performance, alternatives like SiLU and Mish caused a slight degradation. More complex nonlinearities, such as PReLU, Softplus, and CELU, resulted in lower performance. The kernels were also sensitive to size. The 9 × 9 convolution was downsampled to 5 × 5, resulting in a significantly lower error rate. Additional downsampling offers diminishing returns, which highlights the critical importance of an appropriately sized receptive field. The depth effects demonstrate that additional layers to the network increased the feature hierarchies, yielded more accurate results closer to reality without overfitting, and that partial block replication degraded the performance. The experiments of the classifier head revealed that adding extra normalization and dropout layers did not improve generalization, but rather experienced a performance drop for more complicated head structures, implying redundancy instead of improved feature extraction. The initial ConvNeXt model that used Global Average Pooling, with an accuracy of 96.19% and test loss of 0.0609, was used in the pooling analysis. Replacing GAP with Generalized Mean pooling resulted in further improved accuracy (96.97%), however, with a slightly higher test loss of 0.0976, indicating that though GMP would improve feature discrimination, it requires careful tuning to maintain loss stability. However, when GMP was fully integrated and optimized along with other architectural refinements in the proposed ConvGeM-Next structure, accuracy increased to 99.65% with a much lower loss of 0.0133, which proves the effectiveness of architectural enhancements suggested in our method.

**Table 10:** Ablation study analysis.

Model variant	Test accuracy (%)	Test loss	Remarks
Baseline: ConvNeXt (Original, PlantVillage)	96.19	0.0609	Standard architecture
Baseline ConvNext with GMP	96.97	0.0976	Baseline ConvNeXt model with GMP pooling
ConvGem-NeXt (Proposed)	99.65	0.0133	Final proposed model
Activation Functions modifications			
ReLU	97.34	0.0815	Stable baseline
SiLU	94.04	0.1830	Smooth activation
Mish	92.81	0.2100	Non-monotonic
CELU	82.07	0.5422	Unstable gradients
PReLU	76.83	722.0000	Exploding gradients
Softplus	73.92	0.7770	Weak feature activation
Softsign	83.09	0.5080	Saturating activation
Kernel Size Comparison (Conv Layers)			
9×9 Kernel (original)	85.54	0.3200	Baseline receptive field
5×5 Kernel	87.97	0.2800	Reduced spatial context
Architectural Modifications			
Full layer duplication	97.61	0.0520	Enhanced depth
Block-level duplication	94.50	0.1800	Partial redundancy
ConvNeXt Large	95.15	0.1886	Over-parameterized
Custom Classifier Head (FC+BN+ReLU+Dropout)	93.23	0.2223	Improves generalization
3 Flatten Layers + Custom Head	96.15	0.1217	Structured output regularization

Taken together, these findings emphasize that the best balance of accuracy, robustness, and efficiency arises from maintaining ConvNeXt’s original depth with 9×9 kernels, coupled with a lightweight classification head and standard ReLU activation. This configuration consistently provided stable convergence and strong generalization, thereby justifying the final architectural design adopted in this work.

### 4.7 Comparison with deep learning models

This experiment was conducted to assess the efficacy of our ConvGeM-NeXt model against the existing deep learning models, including convolutional and transformer-based architectures that have been widely used for plant disease detection. The comprehensive results of this comparative analysis are presented in **Table 11**, highlighting the performance across all evaluated models. Our ConvGeM-NeXt model achieved a high accuracy of 99.65%, macro precision of 0.9951, macro F1-score of 0.9959, and a minimum test loss of 0.0133 on the PlantVillage dataset. It demonstrated exceptional cross-domain robustness by maintaining 94.69% accuracy, 0.9477 macro precision, and 0.9465 macro F1-score when tested on the more complicated and diverse PlantDoc dataset. In contrast, conventional CNN and transformer models suffered considerable performance declines while transferring from PlantVillage to PlantDoc. For instance, MobileNet-V2 (Ramesh and Hebbar, 2018) and DenseNet-121 (Ferentinos, 2018), which achieved good scores on PlantVillage (98.46% and 99.35%), dropped to 47.56% and 55-58% on PlantDoc. Despite the efficiency of MobileNet-V2 (≈3.4M parameters, 300M FLOPs), its shallow feature extraction limits cross-domain generalization. DenseNet-121, which has deeper connections, is more compute-intensive (≈8M parameters) with longer inference times and, thus, poor suitability for field conditions, which contributes to the poorer performance. A similar pattern was observed with EfficientNet-B0 (Devi et al., 2023), which recorded 99.62% accuracy on PlantVillage but dropped to 57-62% when evaluated under field conditions. EfficientNet-B0 is designed for a parameter efficiency of 5.3 million, but parameter efficiency is not the problem. It is rather a fact that the compound scaling mechanism is unable to capture shifts in domains, thus leading to performance degradation. Poor performance trade-offs are a general characteristic of transformer-based and general approaches. ViT-RoT (Wang et al., 2023) and ResNet-50+CBAM (Zhang et al., 2023) hardly managed 72.85% and 69.52% on PlantDoc, respectively. ResNet-50+CBAM improves attention over spatial cues but incurs additional computation during inference. Even the Swin Transformer (Liu et al., 2022), with its hierarchical attention mechanism, only reached 77.54%, which is still less than our model by over 17% and needs significantly more resources (app. 90M parameters).

**Table 11:** Comparative analysis with deep learning models.

Deep learning models	Accuracy (%)	
	PlantVillage	PlantDoc
Swin Transformer (Liu et al., 2022)	99.12	77.54
ViT-RoT (Wang et al., 2023)	99.86	72.85
ResNet-50 + CBAM (Zhang et al., 2023)	99.15	69.52
EfficientNet-B0 (Devi et al., 2023)	99.62	57–62
DenseNet-121 (Ferentinos, 2018)	99.35	55–58
MobileNet-V2 (Ramesh and Hebbar, 2018)	98.46	47.56
**Proposed ConvGeM-NeXt**	**99.65**	**94.69**

Bold values mean result attained by our model.

These results highlight a consistent limitation among purely convolutional and transformer architectures, where models trained on curated datasets often fail to generalize effectively under real-world visual complexity while also incurring high computational cost. By contrast, the proposed ConvGeM-NeXt method exhibits consistent performance per class, better generalization, and attained an accuracy gain of 15-17% than its competitors on a challenging PlantDoc dataset.

### 4.8 Comparison with state-of-the-art approaches

To ensure a more extensive and fair benchmarking, a comparative analysis was also done on hybrid, lightweight, and recent SOTA architectures, including LeafDoc++ (Richter and Kim, 2025), AgriVision (Wang et al., 2025), DeepLeaf (Pacal et al., 2024), and RF HOG SVM (Pantazi et al., 2019), as shown in **Table 12**. The inclusion of these different paradigms gave a balanced evaluation of the different categories of algorithms as well as the differences in adaptability, robustness, and computational demands. The dual-branch attention network is low-resource (approximation of 12M parameters) with 99.1% and 88.9% accuracy on PlantVillage and PlantDoc, respectively. LeafDoc++ achieved an accuracy of 99.1% on PlanttVillage and 88.9% on PlantDoc. AgriVision, a vision-language fusion framework, achieved 97.8% and 90.1% on PlantVillage and PlantDoc, respectively, but uses an extensive amount of transformer encoders and GPU memory, making it a resource-intensive framework. DeepLeaf, an ensemble-based model that combines CNN and attention fusion, provided 99.4% and 85.6% accuracy on PlantVillage and PlantDoc, respectively, but the multi-branch design and redundant feature aggregation are the causes of DeepLeaf’s slow inference. The RF-HOG-SVM approach is a considerably lightweight method and achieved an accuracy of 95.2% on PlantVillage and 72.4% on PlantDoc. The computational limitations arose due to the lack of representational learning, which affects scalability.

**Table 12:** Comparative analysis with recent state-of-the-art approaches.

Plant disease detection approaches	Accuracy (%)	
	PlantVillage	PlantDoc
LeafDoc++(Lightweight DL) (Richter and Kim, 2025)	99.1	88.9
RF-HOG-SVM (Pantazi et al., 2019)	95.2	72.4
AgriVision(Vision–Language) (Wang et al., 2025)	97.8	90.1
DeepLeaf (Ensemble DL) (Pacal et al., 2024)	99.4	85.6
**Proposed ConvGeM-NeXt**	**99.65**	**94.69**

Bold values mean result attained by our model.

The comparative results demonstrate the effectiveness of our architecture, which combines spatial and spectral feature pattern extraction with a convolutional-transformer employing GMP to improve feature aggregation efficiency while preserving lightweight computational complexity. ConvGeM-NeXt outperformed the most recent SOTA approaches with a margin of 4.6%-7% accuracy. The model not only achieved excellent results on the controlled settings dataset of PlantVillage, but also sustained reliable performance on the real-world imagery dataset of Plantdoc, making it well-suited for practical deployment.

**Table 13** further compares the proposed ConvGeM-NeXt model with representative lightweight, convolutional, and transformer-based architectures in terms of parameter size, computational runtime, and real-world performance on the PlantDoc dataset. Lightweight models such as MobileNet-V2 and EfficientNet-B0 exhibit low computational cost but suffer from severe performance degradation under field conditions. Transformer-based models, while improving accuracy, incur substantially higher computational overhead. In contrast, ConvGeM-NeXt achieves the highest PlantDoc accuracy (94.69%) while maintaining a moderate runtime profile on TPU hardware, demonstrating a favorable balance between model capacity, computational efficiency, and cross-domain generalization.

**Table 13:** Parameter size and computational cost comparison of different models.

Model	Params (M)	Relative runtime	PlantDoc Accuracy (%)
MobileNet-V2	3.4	Very Low	47.56
EfficientNet-B0	5.3	Low	57–62*
DenseNet-121	8.0	Medium	55–58*
ResNet-50 + CBAM	25.6	Medium–High	69.52
Swin Transformer-Small	~50	High	77.54
**ConvGeM-NeXt (Ours)**	**~90M**	**Moderate (TPU-optimized)**	**94.69**

Bold values mean result attained by our model.

### 4.9 Cross-corpus evaluation

This experiment was designed to test the cross-dataset generalizability of the proposed model by examining its performance under various training and testing conditions within and across several domains to investigate its robustness under curated and real-world domains. The experimental design has two transfer scenarios: in the first transfer scenario, the method was trained on the controlled PlantVillage dataset and tested on PlantDoc without any fine-tuning, and in the second, it was trained on PlantDoc and tested on PlantVillage. To provide an unbiased assessment, Top-1 accuracy, macro precision, and macro F1-score were employed to capture both overall and class-balanced generalization. The results also show a clear difference between the two transfer settings. When trained on the PlantVillage and tested on PlantDoc, the model yielded an accuracy of 42.0%, while training on PlantDoc and testing on PlantVillage resulted in 53.23% accuracy (**Table 14**). These findings imply that models trained on PlantVillage lack exposure to the diverse lighting, background, and noise variations present in field data, resulting in weaker cross-domain generalization to real-world agricultural environments. By contrast, even though smaller, models trained using PlantDoc gain some robustness since they are learned on noisy heterogeneous examples and hence learn to adjust in the controlled dataset. These findings show that achieving real-world reliability in agricultural applications requires a balanced combination of clean and noisy training data, supporting stronger domain transfer and better adaptation to natural variations. A detailed per-class evaluation for both transfer scenarios, model trained on PlantVillage and tested on PlantDoc, and vice versa, is presented in **Table 15** and **16**, respectively, highlighting the specific challenges each domain imposes on cross-dataset generalization.

**Table 14:** Cross-dataset generalization performance of the proposed model under train–test domain shift between PlantVillage and PlantDoc datasets.

Train - Test	Accuracy (%)	Macro Precision	Macro F1
PlantVillage - PlantDoc	41.00	0.2238	0.1729
PlantDoc - PlantVillage	53.23	0.5623	0.4685

**Table 15:** Class-wise metrics for the model trained on PlantVillage and tested on PlantDoc.

Class	Precision (%)	Recall (%)	F1-score (%)	Support
apple_healthy	0.00	0.00	0.00	9
apple_rust	0.00	0.00	0.00	10
apple_scab	0.00	0.00	0.00	10
blueberry_healthy	23.00	64.0	34.00	11
cherry_healthy	0.00	0.00	0.00	10
corn_gray_leaf_spot	17.00	25.00	0.20	4
corn_leaf_blight	50.00	42.00	45.00	12
corn_rust	67.00	20.00	31.00	10
grape_black_rot	27.00	38.00	32.00	8
grape_healthy	54.00	58.00	56.00	12
peach_healthy	0.00	0.00	0.00	9
pepper_bacterial_spot	16.00	67.00	26.00	9
pepper_healthy	12.00	75.00	21.00	8
potato_early_blight	17.00	7.000	10.00	14
potato_late_blight	50.00	12.00	20.00	8
raspberry_healthy	0.00	0.00	0.00	7
soybean_healthy	50.00	12.00	20.00	8
squash_powdery_mildew	67.00	33.00	44.00	6
strawberry_healthy	50.00	0.12	20.00	8
tomato_bacterial_spot	0.00	0.00	0.00	9
tomato_early_blight	44.00	44.00	44.00	9
tomato_healthy	0.00	0.00	0.00	8
tomato_late_blight	11.00	60.00	18.00	10
tomato_leaf_mold	0.00	0.00	0.00	6
tomato_mosaic_virus	0.00	0.00	0.00	10
tomato_septoria_leaf_spot	50.00	17.00	25.00	12
tomato_yellow_leaf_curl_virus	0.00	0.00	0.00	15

**Table 16:** Class-wise metrics for the model trained on PlantDoc and tested on PlantVillage. Support indicates the number of test samples per class.

Class	Precision (%)	Recall (%)	F1-score (%)	Support
Apple_healthy	40.00	76.00	53.00	164
Apple_rust	81.00	79.00	80.00	28
Apple_scab	39.00	92.00	55.00	63
Blueberry_healthy	45.00	46.00	46.00	151
Cherry_healthy	25.00	2.00	4.00	86
Corn_gray_leaf_spot	85.00	21.00	34.00	52
Corn_leaf_blight	62.00	70.00	65.00	99
Corn_rust	73.00	91.00	81.00	120
Grape_black_rot	84.00	87.00	85.00	118
Grape_healthy	64.00	98.00	77.00	43
Peach_healthy	21.00	14.00	17.00	36
Pepper_bacterial_spot	51.00	38.00	44.00	100
Pepper_healthy	54.00	72.00	61.00	148
Potato_early_blight	19.00	93.00	32.00	100
Potato_late_blight	12.00	38.00	18.00	100
Raspberry_healthy	100.00	61.00	75.00	38
Soybean_healthy	88.00	38.00	53.00	509
Squash_powdery_mildew	86.00	98.00	92.00	184
Strawberry_healthy	94.00	37.00	53.00	46
Tomato_bacterial_spot	27.00	16.00	21.00	213
Tomato_early_blight	22.00	6.00	9.00	100
Tomato_healthy	100.00	1.00	2.00	159
Tomato_late_blight	62.00	27.00	38.00	191
Tomato_leaf_mold	52.00	42.00	46.00	96
Tomato_mosaic_virus	0.00	0.00	0.00	0
Tomato_septoria_leaf_spot	44.00	34.00	38.00	178
Tomato_yellow_leaf_curl_virus	88.00	84.00	86.00	536

#### 4.9.1 Error case analysis

To analyze the limitations of the ConvGeM-NeXt model under domain shift, we performed error case analysis using per-class precision, recall, and F1-scores from both cross-dataset transfer settings, and results are reported in **Table 17**. Healthy leaf classes were the most frequent source of errors. When the model was trained on PlantVillage and tested on PlantDoc, recall collapsed for *cherry_healthy* (0.02), *tomato_healthy* (0.01), and *peach_healthy* (0.14). In the reverse transfer setting, a near-zero recall was observed for most healthy classes, such as *apple_healthy, cherry_healthy, peach_healthy*, and *tomato_healthy*. These findings show a significant dependence on disease-specific textures, with minimal resistance to changes in illumination or background. Morphologically similar diseases also contributed significantly to errors, particularly *potato early blight vs. potato late blight* and *tomato early blight vs. tomato late blight, which* demonstrated substantial misclassification. In the case of training with PlantDoc and evaluating on the PlantVillage, recall for potato early blight reaches 0.93, while recall for potato late blight drops to 0.38, highlighting the challenges in fine-grained lesion discrimination. Rare-class failures are also prominent due to data scarcity when transferring across domains. Classes such as *tomato mosaic virus* (0.00 recall) and *peach_healthy* (36 samples, recall 0.14) collapse entirely when the model was trained on PlantVillage and tested on PlantDoc. In the reverse setting, multiple low-support categories again exhibit zero recall. Additionally, class imbalance also influenced the performance of the model. Classes such as *soybean_healthy* (509 samples), despite a large sample size achieves only 0.38 recall when trained on PlantDoc. While visually distinctive disease classes such as *tomato_yellow_leaf_curl_virus* retain high recall (0.84), indicating that strong texture cues dominate the learned representations. These findings show that while strong performance under in-domain evaluation, the model limits its generalization ability under domain shift, particularly for healthy leaves, rare diseases, and visually overlapping disease categories.

**Table 17:** Worst performing classes under cross-dataset evaluation.

Transfer Setting (Test- train)	Class Name	Precision (%)	Recall (%)	F1-score (%)
PlantVillage - PlantDoc	apple_healthy	0.00	0.00	0.00
PlantVillage - PlantDoc	cherry_healthy	0.00	0.00	0.00
PlantVillage - PlantDoc	tomato_healthy	0.00	0.00	0.00
PlantDoc - PlantVillage	cherry_healthy	25	02	04
PlantDoc - PlantVillage	tomato_healthy	100	01	02
PlantDoc - PlantVillage	tomato_early_blight	22	06	09

## 5 Conclusion

This paper has presented a robust plant disease classification framework that delivers consistent performance on field and curated datasets. Using the ConvNeXt-Base backbone, our model achieved consistent generalization under varied lighting, cluttered backgrounds, and overlapping symptoms. Comparative analysis against recent CNN and transformer-based models also justified the effectiveness of our model, especially on PlantDoc, where other models showed substantial accuracy decline. These findings demonstrate our model’s ability to balance accuracy, robustness, and generalization, making it a promising solution for real-world agricultural decision-support systems. Despite these results, there are still certain limitations, such as the challenges of highly similar classes of symptoms, biased datasets, and mobile or edge deployment. Future work will focus on developing lightweight hybrid models, attention-guided pruning, and incorporating hardware-aware optimization to enable scalable field deployment.

## Data Availability

Publicly available datasets were analyzed in this study. This data can be found here: https://www.kaggle.com/datasets/abdallahalidev/plantvillage-dataset https://github.com/pratikkayal/PlantDoc-Dataset.

## References

[B1] Abade A. Ferreira P. A. Vidal F. de B. (2021). Plant diseases recognition on images using convolutional neural networks: A systematic review. Comput. Electron. Agric. 185, 106125. doi: 10.1016/j.compag.2021.106125, PMID: 38826717

[B2] Aboelenin S. Elbasheer F. A. Eltoukhy M. M. Farag A. A. Elhosseini A. A. Younis A. . (2025). A hybrid Framework for plant leaf disease detection and classification using convolutional neural networks and vision transformer. Complex Intell. Syst. 11, 142. doi: 10.1007/s40747-024-01764-x, PMID: 30311153

[B3] Agarwal M. Singh A. Arjaria S. Sinha A. Gupta S. (2020). ToLeD: Tomato leaf disease detection using convolution neural network. Proc. Comput. Sci. 167, 293–301. doi: 10.1016/j.procs.2020.03.225, PMID: 38826717

[B4] Ahmad W. Adnan Shah S. M. Irtaza A. (2020). Plants disease phenotyping using quinary patterns as texture descriptor. KSII Trans. Internet Inf. Syst. 14, 3090–3107. doi: 10.3837/tiis.2020.08.009

[B5] Ahmed I. Yadav P. K. (2023). A systematic analysis of machine learning and deep learning based approaches for identifying and diagnosing plant diseases. Sustain. Operations Comput. 4, 96–104. doi: 10.1016/j.susoc.2023.03.001, PMID: 38826717

[B6] Albattah W. Masood M. Javed A. Nawaz M. Albahli S. (2023). Custom CornerNet: a drone-based improved deep learning technique for large-scale multiclass pest localization and classification. Complex Intelligent Syst. 9, 1299–1316. doi: 10.1007/s40747-022-00847-x, PMID: 30311153

[B7] Argüeso D. Picon A. Irusta U. Medela A. San-Emeterio M. G. Bereciartua A. . (2020). Few-Shot Learning approach for plant disease classification using images taken in the field. Comput. Electron. Agric. 175, 105542. doi: 10.1016/j.compag.2020.105542, PMID: 38826717

[B8] Batool A. Hyder S. B. Rahim A. Waheed N. Asgha M. A. (2020). “ Classification and identification of tomato leaf disease using deep neural network,” in Proceedings of the 2020 International Conference on Engineering and Emerging Technologies (ICEET). (New York, NY, USA: IEEE), 1–6.

[B9] Devi R. S. Kumar V. R. Sivakumar P. (2023). EfficientNetV2 model for plant disease classification and pest recognition. Comput. Syst. Sci. Eng. 45.

[B10] Dolatabadian A. Neik T. X. Danilevicz M. F. Upadhyaya S. R. Batley J. Edwards D. (2025). Image-based crop disease detection using machine learning. Plant Pathol. 74 no. 1, 18–38. doi: 10.1111/ppa.14006, PMID: 40046247

[B11] Ferentinos K. P. (2018). Deep learning models for plant disease detection and diagnosis. Comput. Electron. Agric. 145, 311–3185. doi: 10.1016/j.compag.2018.01.009, PMID: 38826717

[B12] Geetharamani G. A. P. J. Pandian A. (2019). Identification of plant leaf diseases using a nine-layer deep convolutional neural network. Comput. Electrical Eng. 76, 323–338. doi: 10.1016/j.compeleceng.2019.04.011, PMID: 38826717

[B13] Kalpana P. Anandan R. Hussien A. G. Alrashoud M. Almutairi S. Alharbi A. . (2024). Plant disease recognition using residual convolutional enlightened Swin transformer networks. Sci. Rep. 14, 8660. doi: 10.1038/s41598-024-56393-8, PMID: 38622177 PMC11018742

[B14] Kuricheti G. Supriya P. (2019). “ Computer vision based turmeric leaf disease detection and classification: a step to smart agriculture,” in Proceedings of the 2019 3rd International Conference on Trends in Electronics and Informatics (ICOEI). (New York, NY, USA: IEEE), 545–549.

[B15] Le Vi N. T. Ahderom S. Apopei B. Alameh K. (2020). A novel method for detecting morphologically similar crops and weeds based on the combination of contour masks and filtered Local Binary Pattern operators. GigaScience 9, giaa017. doi: 10.1093/gigascience/giaa017, PMID: 32129847 PMC7055473

[B16] Lewandowski I. Gaudet N. Lask J. Maier J. Tchouga B. Vargas-Carpintero R. (2017). “ Context,” in Bioeconomy: Shaping the transition to a sustainable, biobased economy ( Springer International Publishing, Cham), 5–16.

[B17] Liu W. Zhang A. (2025). Plant disease detection algorithm based on efficient swin transformer. Computers Materials Continua 82, 3045–3068. doi: 10.32604/cmc.2024.058640

[B18] Liu Z. Mao H. Wu C.-Y. Feichtenhofer C. Darrell T. Xie S. (2022). “ A convnet for the 2020s,” in Proceedings of the IEEE/CVF Conference on CVPR. (New York, NY, USA: IEEE), 11976–11986.

[B19] Mohanty S. P. Hughes D. P. Salathé M. (2016). Using deep learning for image-based plant disease detection. Front. Plant Sci. 7, 215232. doi: 10.3389/fpls.2016.01419, PMID: 27713752 PMC5032846

[B20] Nawaz M. Nazir T. Javed A. Masood M. Rashid J. Kim J. . (2022). A robust deep learning approach for tomato plant leaf disease localization and classification. Sci. Rep. 12, 18568. doi: 10.1038/s41598-022-21498-5, PMID: 36329073 PMC9633769

[B21] Pacal I. Işık G. (2025). Utilizing convolutional neural networks and vision transformers for precise corn leaf disease identification. Neural Computing Appl. 37, 2479–2496. doi: 10.1007/s00521-024-10769-z, PMID: 30311153

[B22] Pacal I. Kunduracioglu I. Alma M. H. Deveci M. Kadry S. Nedoma J. . (2024). A systematic review of deep learning techniques for plant diseases. Artif. Intell. Rev. 57, 304. doi: 10.1007/s10462-024-10944-7, PMID: 30311153

[B23] Pantazi X. E. Moshou D. Tamouridou A. A. (2019). Automated leaf disease detection in different crop species through image features analysis and One Class Classifiers. Comput. Electron. Agric. 156, 96–104. doi: 10.1016/j.compag.2018.11.005, PMID: 38826717

[B24] PlantVillage Dataset. Available online at: https://www.kaggle.com/datasets/abdallahalidev/plantvillage-dataset (Accessed October 01, 2025).

[B25] PlantDoc Dataset. Available online at: https://github.com/pratikkayal/PlantDoc-Dataset (Accessed October 01, 2025).

[B26] Radenović F. Tolias G. Chum O. (2019). “ Fine-Tuning CNN Image Retrieval with No Human Annotation,” in IEEE Transactions on Pattern Analysis and Machine Intelligence, (New York, NY, USA: IEEE), vol. 41, 1655–1668. doi: 10.1109/TPAMI.2018.2846566, PMID: 29994246

[B27] Ramesh S. Hebbar R. (2018). “ Plant disease detection using machine learning,” in Proceedings of the 2018 International Conference on Design Innovations for 3Cs Compute Communicate Control (ICDI3C). (New York, NY, USA: IEEE), 41–45.

[B28] Richey B. Majumder S. Shirvaikar M. Kehtarnavaz N. (2020). “ Real-time detection of maize crop disease via a deep learning-based smartphone app,” in Real-time image processing and deep learning, vol. 11401. (Bellingham, WA, USA: SPIE), 23–29.

[B29] Richter D. J. Kim K. (2025). Assessing the performance of domain-specific models for plant leaf disease classification: a comprehensive benchmark o transfer-learning on open datasets. Sci. Rep. 15, 18973. doi: 10.1038/s41598-025-03235-w, PMID: 40447674 PMC12125250

[B30] Ritharson P.I. Raimond K. Anitha Mary X. Robert J. E. (2024). DeepRice: A deep learning and deep feature based classification of Rice leaf disease subtypes. Artif. Intell. Agric. 11, 34–49. doi: 10.1016/j.aiia.2023.11.001, PMID: 38826717

[B31] Sahu P. K. Jayalakshmi K. Tilgam J. Gupta A. Nagaraju Y. Kumar A. . (2022). ROS generated from biotic stress: Effects on plants and alleviation by endophytic microbes. Front. Plant Sci. 13, 10429365. doi: 10.3389/fpls.2022.1042936, PMID: 36352882 PMC9638130

[B32] Shoaib M. Shah B. Ei-Sappagh S. Ali A. Ullah A. Alenezi F. . (2023). An advanced deep learning models-based plant disease detection: A review of recent research. Front. Plant Sci. 14, 1158933. doi: 10.3389/fpls.2023.1158933, PMID: 37025141 PMC10070872

[B33] Sun Y. Jiang Z. Zhang L. Dong W. Rao Y. (2019). SLIC_SVM based leaf diseases saliency map extraction of tea plant. Comput. Electron. Agric. 157, 102–109. doi: 10.1016/j.compag.2018.12.042, PMID: 38826717

[B34] Sunil C. K. Jaidhar C. D. Patil N. (2023). Systematic study on deep learning-based plant disease detection or classification. Artif. Intell. Rev. 56, 14955–15052. doi: 10.1007/s10462-023-10517-0, PMID: 30311153

[B35] Wang S. Xu D. Liang H. Bai Y. Li X. Zhou J. . (2025). Advances in deep learning applications for plant disease and pest detection: A review. Remote Sens. 17, 698. doi: 10.3390/rs17040698, PMID: 30654563

[B36] Wang C. Zhang J. He J. Luo W. Yuan X. Gu L. (2023). A two-stream network with complementary feature fusion for pest image classification. Eng. Appl. Artif. Intell. 124, 106563. doi: 10.1016/j.engappai.2023.106563, PMID: 38826717

[B37] Wu X. Fan X. Luo P. Choudhury S. D. Tjahjadi T. Hu C. (2023). From laboratory to field: Unsupervised domain adaptation for plant disease recognition in the wild. Plant Phenomics 5, 0038. doi: 10.34133/plantphenomics.0038, PMID: 37011278 PMC10059679

[B38] Yilmaz E. Bocekci S. C. Safak C. Yildiz K. (2025). Advancements in smart agriculture: A systematic literature review on state-of-the-art plant disease detection with computer vision. IET Comput. Vision 19, e70004. doi: 10.1049/cvi2.70004

[B39] Zhang X. Li D. Liu X. Sun T. Lin X. Ren Z. (2023). Research of segmentation recognition of small disease spots on apple leaves based on hybrid loss function and CBAM. Front. Plant Sci. 14, 1175027. doi: 10.3389/fpls.2023.1175027, PMID: 37346136 PMC10279884

[B40] Zhang Y. Song C. Zhang D. (2020). Deep learning-based object detection improvement for tomato disease. IEEE Access 8, 56607–56614. doi: 10.1109/ACCESS.2020.2982456, PMID: 25079929

